# Decision Trees for Managing Impaired Physical Mobility in Multiple Trauma Patients

**DOI:** 10.1111/jan.17010

**Published:** 2025-05-07

**Authors:** Raisa Camilo Ferreira, Karen Dunn‐Lopez, Sue Moorhead, Anna Krupp, Bruna Valentina Zuchatti, Luciana Aparecida Costa Carvalho, Micneias Tatiana de Souza Lacerda Botelho, Erika Christiane Marocco Duran

**Affiliations:** ^1^ School of Nursing State University of Campinas Campinas Brazil; ^2^ College of Nursing University of Iowa Iowa City IA USA; ^3^ School of Nursing Universidade Federal de Mato Grosso Sinop Brazil

**Keywords:** artificial intelligence, clinical decision‐making, decision trees, electronic health records, multiple trauma, standardised nursing terminology

## Abstract

**Aim:**

To develop and validate decision trees using conditional probabilities to identify the predictors of mortality and morbidity deterioration in trauma patients.

**Design:**

A quasi‐experimental longitudinal study conducted at a Level 1 Trauma Center in São Paulo, Brazil.

**Method:**

The study analysed 201 patient records using standardised nursing documentation (NANDA International and Nursing Outcomes Classification). Decision trees were constructed using the chi‐squared automatic interaction detection (CHAID) algorithm and validated through K‐fold cross‐validation to ensure model reliability.

**Results:**

Decision trees identified key predictors of survival and mobility deterioration. Patients who did not require (NOC 0414) *Cardiopulmonary Status* but required (NOC 0210) *Transfer Performance* had a 97.4% survival rate. Conversely, those requiring (NOC 0414) *Cardiopulmonary Status* had a 25% risk of worsening mobility, compared to 9% for those who did not. *K‐fold cross‐validation* confirmed the model's predictive accuracy, reinforcing the robustness of the decision tree approach (Value).

**Conclusion:**

Decision trees demonstrated strong predictive capabilities for mobility outcomes and mortality risk, offering a structured, data‐driven framework for clinical decision‐making. These findings underscore the importance of early mobilisation, tailored rehabilitation interventions and assistive devices in improving patient recovery. This study is among the first to apply decision trees in this context, highlighting its novelty and potential to enhance trauma critical care practices.

**Implications for the Profession and/or Patient Care:**

This study highlights the potential of decision trees, a supervised machine learning method, in nursing practice by providing clear, evidence‐based guidance for clinical decision‐making. By enabling early identification of high‐risk patients, decision trees facilitate timely interventions, reduce complications and support personalised rehabilitation strategies that enhance patient safety and recovery.

**Impact:**

This research addresses the challenge of improving outcomes for critically ill and trauma patients with impaired mobility by identifying effective strategies for early mobilisation and rehabilitation. The integration of artificial intelligence‐driven decision trees strengthens evidence‐based nursing practice, enhances patient education and informs scalable interventions that reduce trauma‐related complications. These findings have implications for healthcare providers, rehabilitation specialists and policymakers seeking to optimise trauma care and improve long‐term patient outcomes.

**Patient or Public Contribution:**

Patients provided authorisation for the collection of their clinical data from medical records during hospitalisation.

## 1 Introduction

Trauma, or physical injury, is a leading cause of death and disability, particularly among individuals under 40 years old (Benhamed et al. 2023). Each year, over 50 million people sustain injuries, making this a critical global health issue. Injuries—whether due to violence, road traffic accidents or falls—place a substantial burden on national economies, reduce productivity and profoundly affect an individual's quality of life and overall well‐being (Benhamed et al. 2023; Samoborec et al. 2018; Vos et al. 2020). Beyond the immediate physical consequences, trauma can also lead to long‐term psychological distress and further exacerbate its impact on individuals and society (Benhamed et al. 2023; Samoborec et al. 2018; Vos et al. 2020; Driessen et al. 2021; Skogstad et al. 2014).

Driessen et al. (2021) report that multiple traumas account for approximately 50% of all traumatic events and pose significant challenges due to their complexity and heightened risk of adverse outcomes. The presence of multiple injuries not only increases immediate life‐threatening risks but also complicates management and rehabilitation. These patients often experience prolonged hospitalisations, require extensive surgical interventions and undergo intensive rehabilitation to regain functional independence. The risk of poor outcomes is further exacerbated by the severity and combination of injuries, the potential for delayed complications and the complexity of treatment regimens (Benhamed et al. 2023; Samoborec et al. 2018).

Despite advancements in trauma care and rehabilitation, multiple traumas remain a major challenge for patients' and healthcare resources (Vos et al. 2020). Nurses, with their consistent bedside presence and holistic care approach, are uniquely positioned to meet the complex needs of trauma patients (Ferreira et al. 2024). In this sense, effective clinical decision‐making involves collecting, interpreting and evaluating data based on scientific evidence, which is central to nursing practice (Nibbelink and Brewer 2018). Internationally recognised as crucial, decision‐making skills and clinical judgement are essential for managing trauma care, improving patient outcomes and optimising healthcare resources (Nibbelink and Brewer 2018). Additionally, nurses navigate complex factors to meet diverse patient and family needs, balancing patient assessments, treatment options, family preferences and available resources (Nibbelink and Brewer 2018). They manage multiple patients and coordinate care across fragmented Electronic Health Record (EHR) data (Ferreira et al. 2024; Nibbelink and Brewer 2018). This demands keen clinical judgement, effective prioritisation and adaptability to ensure comprehensive, accurate and compassionate care for each patient (Nibbelink and Brewer 2018).

Recent advances in technology and health information technology have transformed data collection and analysis and its application in nursing (Junaid et al. 2022). These advancements provide today's nurses with unparalleled opportunities to leverage data‐driven insights, empowering them to make informed decisions that positively impact patient care. As nurses increasingly engage with digital health records and other technologies, the potential for optimising patient outcomes through data‐driven approaches continues to expand clinical judgement (Nibbelink and Brewer 2018; Junaid et al. 2022).

## 2 Background and Significance

Over 85% of multiple trauma patients are anticipated to have the nursing diagnosis NANDA International (NANDA‐I) ‘Impaired Physical Mobility’ (00085) (Herdman et al. 2021; Ferreira and Duran 2019). This nursing diagnosis is associated with poor patient outcomes, complications and significantly impacts the recovery trajectory and overall quality of life of those affected. affected (Ferreira et al. 2024; Ferreira and Duran 2019) Standardised nursing terminologies (SNTs) have been used by nurses since the 1970s (Monsen et al. 2023) to document care, thus serving as a reliable and valid way to assess nurses' impact on patient outcomes (Bertocchi et al. 2023). SNTs cover nursing diagnoses, interventions and outcomes, enabling the capture of the nursing care process. Over time, these terminologies have been refined to enhance their utility in practice, research and policy, ensuring that nursing's contributions are well‐documented and measurable across various settings (Fennelly et al. 2021).

To further enhance nursing practice and improve patient outcomes, technological innovations are increasingly being integrated into healthcare. Artificial intelligence (AI) offers a promising solution to improve patient outcomes by enhancing a healthcare professional's expertise through assisting in the analysis of complex data, identifying patterns and predicting outcomes. AI enables more accurate diagnoses, personalised treatments and improved efficiency in healthcare systems by reducing unnecessary tests and ineffective treatments (Delebarre et al. 2022; Vera‐Salmerón et al. 2022). By structuring historical and current data, AI supports informed, personalised decision‐making. For instance, AI can identify critical survival factors for multiple trauma patients, predict outcomes and prioritise urgent interventions, thereby improving diagnostic accuracy and resource management (Junaid et al. 2022; Delebarre et al. 2022; Vera‐Salmerón et al. 2022).

Furthermore, developing and validating decision support tools using decision trees (DTs) presents a promising approach for enhancing data‐driven decision‐making in clinical practice. Decision trees are a supervised learning method in machine learning, a subarea of AI, focused on developing algorithms that enable machines to learn and improve from data autonomously. They help to structure data, identify patterns and predict outcomes to improve AI system accuracy and reliability in clinical settings. Unlike statistical tests, which evaluate hypotheses or best practices that provide broad guidelines, DTs offer a visual and structured method for data decision‐making by mapping out potential decisions and outcomes based on input features (Delebarre et al. 2022; Vera‐Salmerón et al. 2022). Traditional methods often struggle with the complexity of managing impaired mobility in this population, highlighting the need for an innovative, evidence‐based approach (Delebarre et al. 2022; Vera‐Salmerón et al. 2022).

Compared to widely used clinical decision support (CDS) tools, which include algorithms, guideline dashboards and alerts, DTs are a specific type of predictive model that offers an interpretable decision process suitable for real‐time clinical scenarios (Delebarre et al. 2022; Vera‐Salmerón et al. 2022; Mohanasundari et al. 2023; Younas and Durante 2023). Furthermore, in contrast to conventional CDS tools, DTs combine statistical analysis with a visual structure representing decision points and possible outcomes, thus breaking down complex decision‐making processes into simple, visual steps, enhancing the interpretability of data, making it easier for healthcare professionals to make informed, personalised decisions. Each node in a DT represents a decision or test, and branches show outcomes or decisions, facilitating pattern identification and prediction. This approach leverages statistical insights to support accurate, reliable clinical decision‐making (Delebarre et al. 2022; Vera‐Salmerón et al. 2022; Mohanasundari et al. 2023; Younas and Durante 2023).

Although the use of DTs in healthcare holds significant promise, they remain relatively unexplored, especially in supporting nurse decision‐making. DTs can integrate patient symptoms, medical history and other relevant factors to guide clinical decision‐making, enabling early identification of high‐risk patients and facilitating timely interventions (Delebarre et al. 2022; Vera‐Salmerón et al. 2022; Mohanasundari et al. 2023; Younas and Durante 2023). This proactive approach supports a patient‐centred model of care and can be integrated with the NANDA‐I diagnosis Taxonomy (Herdman et al. 2021), Nursing Outcomes Classification (NOC) (Moorhead et al. 2018) and Nursing Interventions Classification (NIC) (Butcher et al. 2019) to streamline documentation and patient management in EHRs, thus enhancing care quality and team communication (Junaid et al. 2022). Therefore, this paper explores the development and validation of DT‐based decision support tools to enhance and support the care of multiple trauma patients, aligning with key international and national AI and machine learning trends (Delebarre et al. 2022; Vera‐Salmerón et al. 2022).

## 3 Aim

To develop and validate decision trees based on conditional probabilities to identify features that predict death and deterioration of mobility.

## 4 Methods

### 4.1 Design

This research uses a quasi‐experimental, longitudinal approach with repeated assessments (Younas and Durante 2023). We adhered to the relevant Enhancing the Quality and Transparency of Health Research (EQUATOR) guidelines for transparent and comprehensive reporting of our study and the Strengthening the Reporting of Observational Studies in Epidemiology (STROBE) guidelines (Supporting Information 1).

### 4.2 Research Location

Data collection was carried out at a Level 1 trauma centre in the rural area of the state of São Paulo, Brazil, from February to August 2021. The patients were cared for in the trauma intensive care units (ICUs), orthopaedic wards, traumatology wards, neurosurgery wards or trauma surgery wards.

### 4.3 Inclusion and Exclusion Criteria

The study included adult patients aged 18–65 years with a diagnostic hypothesis of multiple traumas, according to Berlin criteria (standardised criteria to classify trauma patients using physiological parameters and trauma scores) (Driessen et al. 2021), and Impaired Physical Mobility (00085), identified previously by the nurses or later by the trained research team (Polit and Beck 2022; Botelho et al. 2019). Patients with known motor deficits prior to trauma and those hospitalised for less than 24 h were excluded (Polit and Beck 2022).

### 4.4 Sample

Sample size calculation was based on the formula for estimating a proportion considering a finite population, assuming a proportion of 0.88 (88%; result obtained in the preliminary study) (Ferreira and Duran 2019). A sampling error of 5% and a significance level of 5% (Polit and Beck 2022) were assumed, and the minimum sample size was obtained for 163 patients.

### 4.5 Data Collection Procedures

Data collection was conducted by trained research nurses, who were members of our Care Technologies in Nursing and Health research team. Training, as recommended by Botelho et al. (2019), included workshops focused on the diagnostic category Impaired Physical Mobility (00085) and its associated NOCs in patients with multiple traumas. The data collection process utilised instruments developed by the research team, designed to identify nursing outcomes related to multiple trauma patients and Impaired Physical Mobility (Polit and Beck 2022; Botelho et al. 2019). If the hospital nurse had not assigned the nursing diagnosis of Impaired Physical Mobility, but the trained research nurse identified its presence, the research nurse collaborated with the hospital care team to ensure its inclusion in the patient's care plan.

Physical and EHRs were consulted during data collection. In addition, the research team performed comprehensive analyses, including physical examinations and direct observation of all patients included in the sample. Patients were also classified according to trauma severity (Botelho et al. 2019). The variables used in data collection are outlined in Table 1.

**TABLE 1:** Variables used for data collection.

Variable	Definition	Frequency collected	Variable type	Usage in analysis
Medication	List of medications administered to the patient	Daily	Categorical/Numeric	Mediator variable
Nursing care plans	Detailed nursing care plans for each patient	Daily	Text/Structured	Predictor and independent variables
Vital signs	Measurements of temperature, blood pressure, heart rate and respiratory rate	Hourly/Daily	Numeric	Mediator variable
Laboratory tests	Results of various laboratory tests (e.g., blood tests, imaging)	As needed	Numeric/Categorical	Mediator variable
Data of multiple traumas	List extent of multiple traumas	Upon admission	Text/Structured	Mediator variable
Initial treatments	Treatments administered immediately following trauma	Upon admission	Categorical/Numeric	Predictor variable
Abbreviated Injury Scale (AIS)	Score indicating the severity of individual injuries	Upon admission	Numeric	Mediator variable
Injury Severity Score (ISS)	Composite score of multiple injuries	Upon admission	Numeric	Mediator variable
Revised Trauma Score (RTS)	Score based on physiological response to trauma	Upon admission	Numeric	Mediator variable
Trauma and Injury Severity Score (TRISS)	Predictive score for survival probability	Upon admission	Numeric	Mediator variable
Clinical outcome	Final patient outcome (discharge, transfer, death)	At discharge	Categorical	Dependent variable
Mobility status at discharge	Patient's mobility status at discharge (improved, maintained, worsened)	At discharge	Categorical	Dependent variable
NOC outcomes^a^	Nursing outcomes classified as resolved, worsened, or unchanged	Every 24 h	Categorical^b^	Independent variable

^a^ Each NOC proposed by the research team was evaluated every 24 h and judged as resolved, worsened or unchanged. NOC outcomes consist of a list of indicators. The indicators are measured using a 5‐point Likert scale to evaluate patient progress following the implementation of nursing interventions.

^b^ NOC scales are standardised tools used to measure patient outcomes based on nursing care. Each outcome is assigned a rating on a *Likert‐type* scale (usually from 1 to 5), where 1 represents the least desirable condition and 5 represents the most desirable. These scales allow nurses to assess a patient's status before, during and after interventions. The use of NOC scales provides a reliable and quantifiable method for tracking the effectiveness of nursing interventions and supports evidence‐based practice in clinical settings.

All NOC values collected during the patient's hospitalisation were aggregated to understand the progression of each outcome over time. The main focus of tracking results was whether that NOC (goal) reached a resolved state, as well as any cases where results worsened or remained unchanged (Moorhead et al. 2018). For inducing decision trees, it is necessary to use NOCs that exhibit a prevalence different from 0% or 100% (Shalev‐Shwartz and Ben‐David 2014). Decision trees were developed by identifying patterns in the NOC data, such as final NOC status, patient mobility status and final outcome. These assessments continued until the patient's outcome was determined: discharge, transfer or death. The research utilised the NANDA‐I 12th edition (Herdman et al. 2021) and NOC 6th edition (Moorhead et al. 2018) taxonomies to describe nursing diagnoses and outcomes.

### 4.6 Ethical Considerations

Patient, family members or legal guardians' consent was required before data collection, and the free and informed consent form was signed, agreeing to their participation in the research. The study adhered to all ethical requirements listed in the WMA Declaration of Helsinki and the Brazilian National Health Counseling Resolution. The Institutional Review Board of the State University of Campinas, Brazil, approved this research under opinion number 3.587.784, with the issuance of the ethical appreciation certificate 13529619.3.0000.5404 in the year 2019, as detailed in Supporting Information 3.

### 4.7 Analysis

#### 4.7.1 Descriptive Analysis

Data description was performed using frequencies and percentages for qualitative variables and measures of central tendency (mean, median) and dispersion (standard deviation, interquartile range) for quantitative variables. Normality was assessed using the Shapiro–Wilk test, and non‐normally distributed variables were summarised using medians and interquartile ranges. Statistical analyses were conducted using Statistical Analysis System (SAS) version 9.4 and SPSS version 22.0, with a significance level set at *α* = 0.05 (Polit and Beck 2022).

#### 4.7.2 Association Analyses

To assess associations between categorical variables and the outcomes (‘death’ and ‘worsened outcome’), chi‐squared tests were used when expected cell counts were ≥ 5, and Fisher's exact test was applied when expected frequencies were lower. Associations between complication variables—mechanical restriction, rhabdomyolysis, acute infection and fever—and the outcome of death were analysed. Fisher's exact test was used for rhabdomyolysis (*p* = 0.0036), acute infection (*p* = 0.0027) and fever (*p* = 0.0161), as expected counts were low (Polit and Beck 2022).

#### 4.7.3 Comparison of Trauma Scores

To compare trauma scores (Injury Severity Score [ISS] and Trauma and Injury Severity Score [TRISS]) between outcome groups, the Mann–Whitney *U*‐test was used due to the non‐normal distribution of these variables, as confirmed by the Shapiro–Wilk test. Patients who died had significantly higher ISS (median: 54.71 vs. 33.59, *p* < 0.0001) and lower TRISS scores (median: 34.98 vs. 79.93, *p* < 0.0001). Additionally, ISS was significantly higher in patients with worsened mobility outcomes compared to others (48.41 vs. 33.42, *p* = 0.0002) (Polit and Beck 2022).

#### 4.7.4 Regression Analysis

Poisson regression analysis was used to evaluate the association between ambulation, transfer performance and the selected outcomes: death and improved mobility (NOC). Poisson regression was selected because the outcomes ‘death’ and ‘improved mobility’ were analysed as event counts, meaning they represent the number of occurrences within specific groups rather than binary outcomes. Prevalence ratios (PR) with 95% confidence intervals (CI) were calculated to estimate the effect of independent variables on each outcome. Covariates, including age, ISS and comorbidities, were adjusted in the regression models to account for potential confounders (Polit and Beck 2022).

#### 4.7.5 Induction of Decision Trees

The process of decision tree induction utilised the Chi‐squared Automatic Interaction Detection (CHAID) algorithm. This algorithm enables the analysis and understanding of complex relationships between independent (predictor) variables (NOCs) and a dependent outcome variable within a dataset. In this case, separate decision trees were generated for each outcome (death or worsening of mobility) based on the prevalence provided in the nursing outcomes (NOC) results (labels) as independent variables. CHAID is an algorithm that relies on chi‐squared tests to identify statistically significant relationships between independent variables and the outcome variable. It divided the data into groups based on independent variables to maximise the difference in outcome occurrence rates. As the CHAID algorithm was applied, a decision tree was constructed recursively (Milanović and Stamenković 2016). Starting with the root node representing the entire dataset, nodes were then split based on chi‐squared tests until stopping criteria were met. CHAID selected the best splitting nodes based on criteria such as *p*‐value and statistical significance. The final tree was the one that best exemplified the relationship between the independent variables and the outcome, considering statistical significance. As basic parameters for generation, a significance level for node splitting and merging of categories of 0.05 was determined, with likelihood ratio as the method to obtain the chi‐squared value. Statistical tests were conducted to check for normality and relationships between variables (Shalev‐Shwartz and Ben‐David 2014; Milanović and Stamenković 2016).

#### 4.7.6 K‐Fold Cross‐Validation for Decision Trees

**FIGURE 1:**
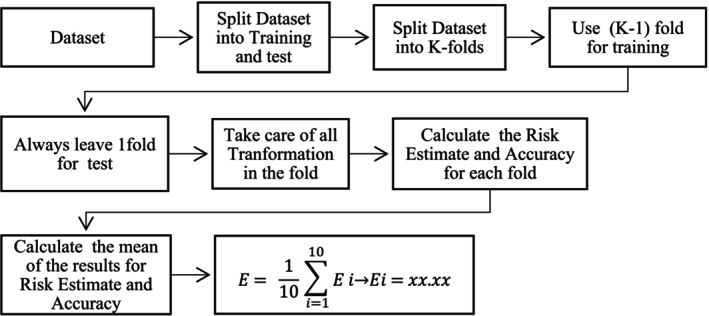
Steps K‐fold cross‐validation for decision trees, adapted from Blockeel and Struyf (2002).

K‐fold cross‐validation was employed to evaluate the performance of decision trees. This technique involves dividing the dataset into *k* subsets, or folds, and iteratively training and evaluating the model times, each time using a different fold as the validation set. This approach allowed for the evaluation of the stability and effectiveness of the decision tree model in predicting outcomes, increasing its credibility for real‐world applications (Shalev‐Shwartz and Ben‐David 2014; Milanović and Stamenković 2016). To perform the analysis, we used the Statistical Package for Social Science (SPSS) version 22.0 software. The steps made by the software are described below in Figure 1.

The K‐fold cross‐validation process will be thoroughly detailed, as executed by the software for decision tree construction. In this method, the dataset was randomly divided into 10 equally (or nearly equally) subsets, known as folds. This number of folds was chosen as it is the default setting in the SPSS software used for the analysis. For each iteration (where ranges from 1 to 10), the model is trained on K‐1 (10–1) folds and tested on the remaining fold, with the test and training subsets alternating. The fold was reserved as the validation set. The remaining nine folds were combined to form the training set. A decision tree model was trained on the training set and subsequently evaluated on the validation set. This process repeats K times (10 times), ensuring all observations are used for both training and testing. The final model performance is determined by averaging the results across all iterations, providing an accurate evaluation of the model's efficacy (Blockeel and Struyf 2002). During each iteration, two key metrics were recorded:

Risk estimates: These provide a measure of the decision tree's error rate, representing the likelihood of incorrect classifications.

$$\text{Risk}=\frac{1}{N}\sum_{i}^{N}\mathrm{II}\left(y_{i}\neq {\hat{y}}_{i}\right)$$

where *Ν* is the total number of instances in the validation set. *y*_*i*_ is the actual class label of instance. ${\hat{y}}_{i}$ is the predicted class label of instance. II (condition) is the indicator function that returns 1 if the condition is true (i.e., $y_{i}\neq {\hat{y}}_{i}$) and 0 otherwise.

Accuracy: This measures the percentage of correct classification of the model, indicating the proportion of correctly classified instances in the validation set.

$$\text{Accuracy}=\frac{1}{N}\sum_{i=1}^{N}\mathrm{II}\left(y_{i}={\hat{y}}_{i}\right)\times 100\%$$

where *Ν* is the total number of instances in the validation set. *y*_*i*_ is the actual class label of instance. ${\hat{y}}_{i}$ is the predicted class label of instance. II (condition) is the indicator function that returns 1 if the condition is true (i.e., $y_{i}={\hat{y}}_{i}$) and 0 otherwise.

The performance metrics obtained from each of the 10 iterations were averaged to provide an overall estimate of the model's performance. This averaged value offers a robust indication of the decision tree's generalisation ability (Milanović and Stamenković 2016).

## 5 Results

### 5.1 Descriptive Analysis

The sample included 201 patients; the majority were male, and the main trauma mechanisms were motorcycle (*n* = 84) 41.79%, car accident (*n* = 23) 11.44%, fall from height (*n* = 23) 11.44%, interpersonal aggression (*n* = 17) 8.45%. More sample characteristics are described below in Table 2.

**TABLE 2:** Characterisation of multiple trauma patients.

Variable	Category	*n*	%
Gender (biologic)	Masculine	180	89.55
	Feminine	21	10.45
Death	No	187	93.03
	Yes	14	6.97
Possibility of mobility recovery	No	38	18.91
	Yes	139	69.15
	Partially	24	11.94
Type of trauma	Blunt	177	88.05
	Penetrating	22	10.95
	Blunt and penetrating	2	1.00
Mobility outcome	Worsened	22	10.95
	Improved	169	84.08
	Sustained	10	4.98

The frequencies, mean, median, standard deviation, minimum and maximum of variables related to the severity, age (years), total length of stay, length of stay in the ICU, length of stay in the wards and trauma scores are presented in Table 3.

**TABLE 3:** Characterisation of patients suffering from multiple traumas.

Variable	Average	Standard deviation	Minimum	Q1	Median	Q3	Maximum
GCS^a^	12.26	3.98	3.00	11.00	15.00	15.00	15.00
Age	37.06	13.21	18.00	26.00	36.00	49.00	65.00
Total length of stay	22.80	30.09	2.00	9.00	14.00	24.00	268.00
ISS^a^	35.06	16.08	17.00	19.00	33.00	48.00	75.00
TRISS^a^	76.79	29.50	1.05	66.98	92.82	98.23	98.67
ICU length of stay	15.51	15.67	0.00	6.00	10.00	19.00	84.00
Wards' length of stay	17.95	24.74	1.00	6.00	11.00	21.00	238.00
MAP^a^	85.53	22.45	0.00	70.00	93.33	96.67	146.00
RR^a^	19.32	5.43	5.00	18.00	18.00	20.00	40.00
HR^a^	94.02	1.53	45.00	81.00	90.50	105.00	161.00
RTS^a^	6.93	1.50	1.02	6.38	7.84	7.84	7.84

Abbreviations: GCS, Glasgow Coma Scale; HR, heart rate; ISS, Injury Severity Score; MAP, Mean arterial pressure; RR, respiratory rate; RTS, Revised Trauma Score; TRISS, Trauma and Injury Severity Score.

^a^ These variables were calculated based on the information collected during the patient's admission.

There were identified associations between complication variables and the outcome of death. The variables include mechanical restriction, rhabdomyolysis, acute infection and fever. Mechanical restriction showed no significant association with death (*p* = 0.7457). However, rhabdomyolysis (*p* = 0.0036), acute infection (*p* = 0.0027) and fever (*p* = 0.0161) showed significant associations with death. For instance, 26.32% of patients with rhabdomyolysis died, compared to 4.42% without it (*p*‐value obtained using Fisher's exact test).

Additionally, we compared the trauma score variables and mobility outcome of death. The ISS and the TRISS were significantly associated with death. Patients who died had a higher average ISS (54.71) compared to those who survived (33.59), with a *p*‐value of < 0.0001. Similarly, the average TRISS for patients who died was 34.98, compared to 79.93 for those who survived, also with a *p*‐value of < 0.0001 (*p*‐value obtained using the Mann–Whitney test). Comparisons were made between trauma scores and the outcomes ‘worsened’ and ‘other’. The ISS was significantly higher for patients with worsened outcomes (average 48.41) compared to others (average 33.42), with a *p*‐value of 0.0002 (*p*‐value obtained using the Mann–Whitney test).

For the clinical validation stage, accuracy measurements, and decision tree induction, 47 NOCs and their indicators were submitted in full. The table below shows the NOC and indicators with their frequencies, as shown in Table 4.

**TABLE 4:** Frequency of nursing outcomes (NOC) related to impaired physical mobility in patients with multiple traumas.

Code	NOC label	*n*	%
0300	Self‐Care: Activities of Daily Living (ADL)	201	100
0301	Self‐Care: Bathing	201	100
0303	Self‐Care: Eating	70	34.83
0305	Self‐Care: Hygiene	201	100
0308	Self‐Care: Oral Hygiene	201	100
0310	Self‐Care: Toileting	201	100
0204	Immobility Consequences: Physiological	201	100
0401	Circulation Status	201	100
1913	Physical Injury Severity	201	100
1104	Bone Healing*	175	87.06
0414	Cardiopulmonary Status*	24	11.94
0415	Respiratory Status	201	100
0911	Neurological Status: Central Motor Control	201	100
0914	Neurological Status: Spinal Sensory/Motor Function	201	100
2402	Sensory Function: Proprioception	0	0
1300	Acceptance: Health Status*	63	31.34
0205	Immobility Consequences: Psycho‐Cognitive*	48	23.88
0909	Neurological Status	201	100
1308	Adaptation to Physical Disability*	6	2.99
1211	Anxiety Level	48	23.88
0900	Cognition	201	100
2109	Discomfort Level	201	100
2102	Pain Level	201	100
1605	Pain Control	201	100
3010	Client Satisfaction: Safety	201	100
1935	Risk Control: Aspiration	0	0
1909	Fall Prevention Behaviour*	186	92.54
0203	Body Positioning: Self‐Initiated	201	100
1637	Musculoskeletal Rehabilitation Participation	201	100
0208	Mobility	201	100
0200	Ambulation*	160	79.60
0210	Transfer Performance*	131	65.17
1616	Body Mechanics Performance	0	0
1811	Knowledge: Prescribed Activity	201	100
1827	Knowledge: Body Mechanics	0	0
0201	Ambulation Wheelchair	0	0
0202	Balance	201	100
0212	Coordinated Movement	201	100
0222	Gait	201	100
0005	Activity Tolerance	0	0
0001	Endurance	0	0
0002	Energy Conservation	0	0
0918	Heedfulness of Affected Side*	86	42.78
3208	Knowledge: Musculoskeletal Rehabilitation	201	100
0210	Transfer Performance*	175	87.06
0211	Skeletal Function*	175	87.06
1101	Tissue Integrity: Skin & Mucous Membranes	201	100
1705	Health Orientation	201	100
2301	Medication Response	201	100
1007	Nutritional Status: Energy	0	0

*Note:* The label NOCs marked with * were used to induce the Decision Trees.

The relationship between NOC Ambulation and NOC Transfer Performance and deaths and worsening outcomes, are shown in Table 5.

**TABLE 5:** Poisson regression with the outcomes.

Dependent variable	Independent variables	Prevalence ratio	CI (95%)		*p*
			Inferior limit	Upper limit	
Death*	Ambulation	0.92	0.88	0.97	0.0017
Improved mobility (NOC)**	Ambulation	0.91	0.84	0.98	0.0167
	Transfer performance	1.16	1.00	1.35	0.0520

Abbreviation: CI, confidence interval.

* The probability of presenting the result ‘No death’ was estimated.

** The probability of presenting the result ‘Improved mobility’ was estimated.

**FIGURE 2:**
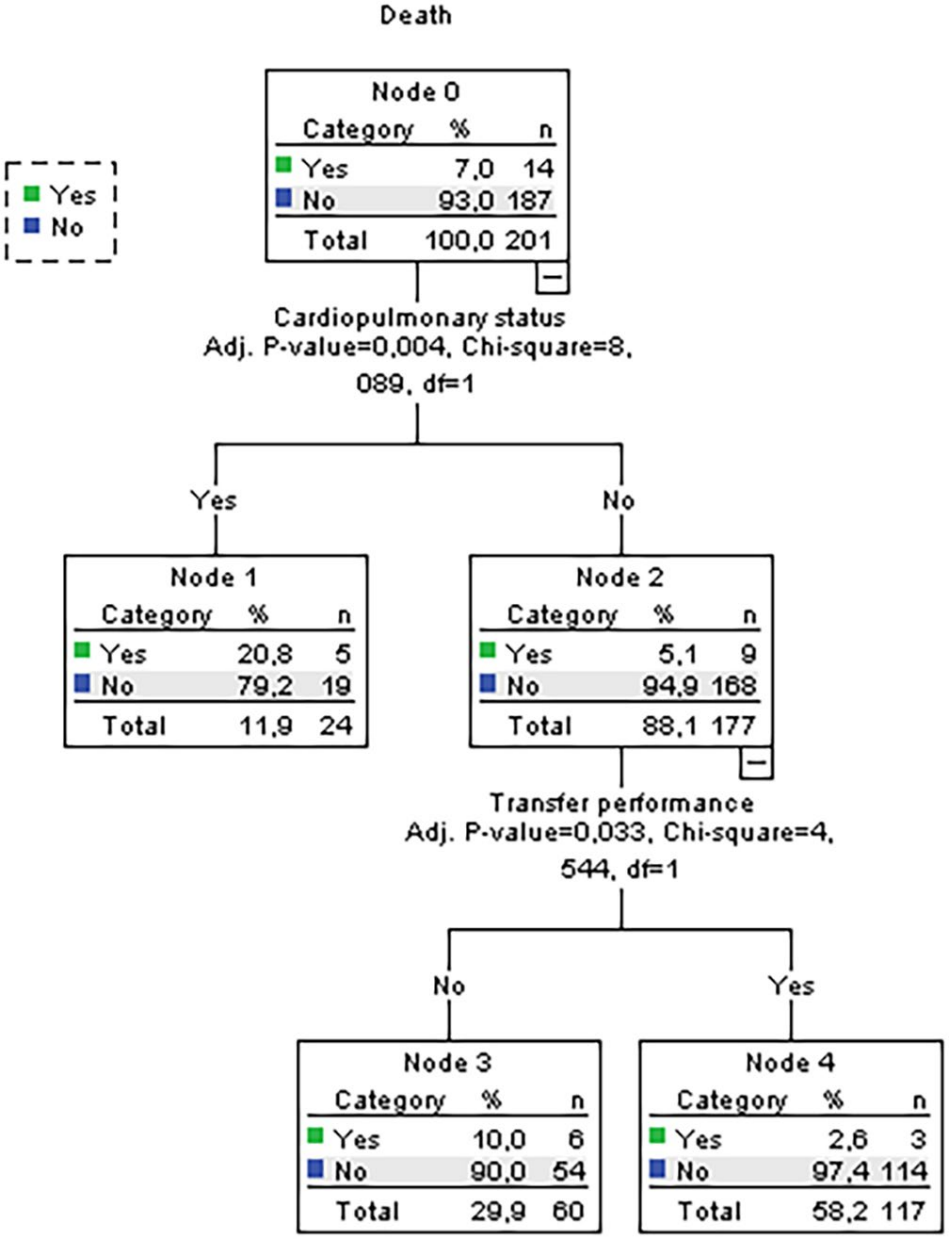
Decision tree for multiple trauma patients with a nursing diagnosis of Impaired Physical Mobility (00085) in relation to the death outcome.

**FIGURE 3:**
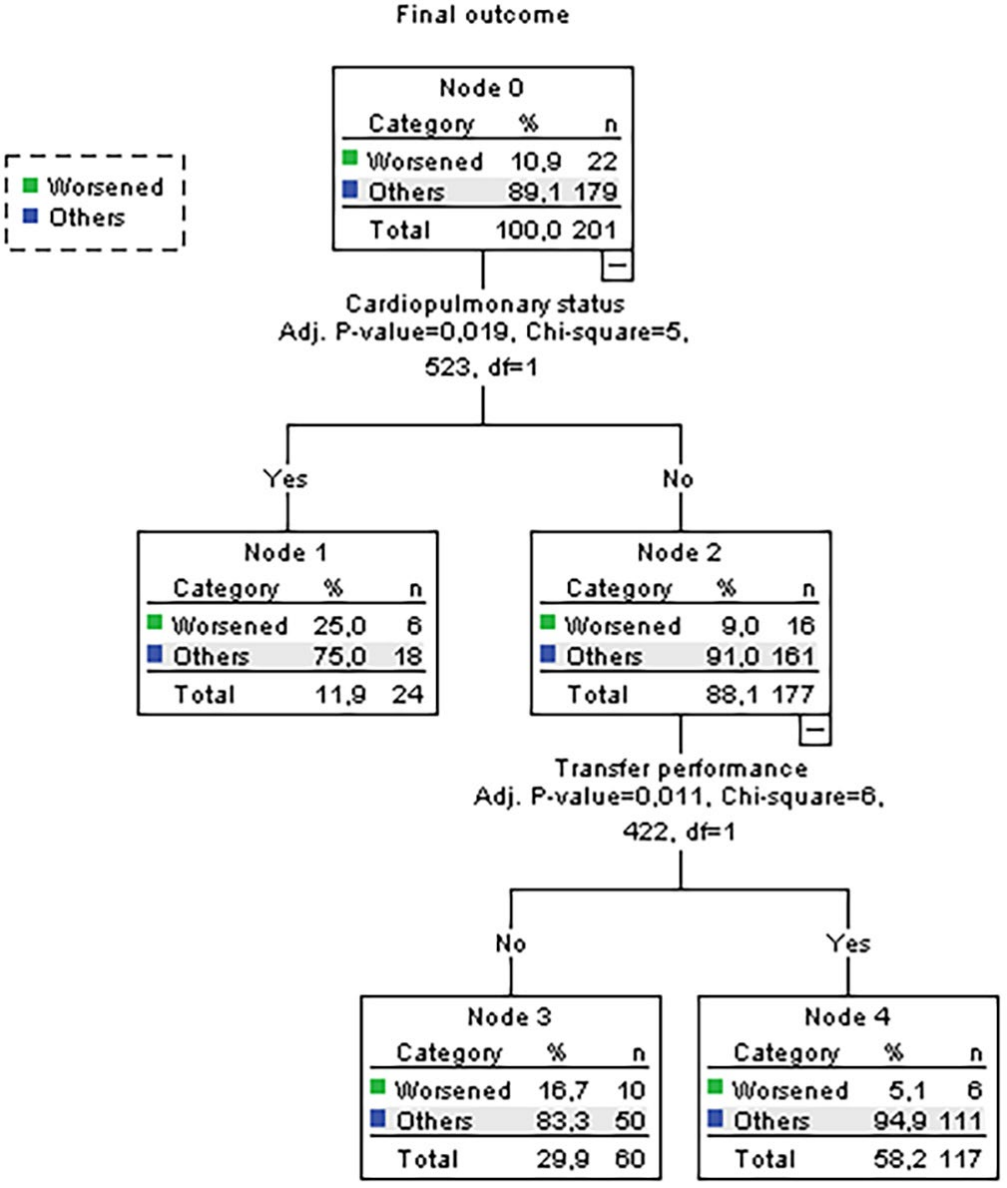
Decision tree for multiple traumas patients with a nursing diagnosis Impaired Physical Mobility (00085), in relation to the worsened mobility outcome.

The decision trees induced by the CHAID algorithm are shown in Figures 2 and 3.

The decision trees evidenced that patients not requiring monitoring for NOC 0414 Cardiopulmonary Status but needing assessment for NOC 0210 Transfer Performance had a 97.4% survival rate, underscoring the strong association between effective transfer performance and reduced mortality. In contrast, patients monitored for NOC 0414 Cardiopulmonary Status had a higher mortality rate (20.8%) compared to those not monitored (10.0%). Among patients with good transfer performance scores (NOC 0210), 94.9% achieved improved or resolved mobility outcomes, compared to only 5.1% with poor scores, highlighting the critical influence of transfer performance on mobility.

Patients receiving mobility interventions had significantly lower mortality rates (2.6%) compared to those who did not (10.0%) with statistical significance (adjusted *p*‐value = 0.033). Additionally, patients assessed for NOC 0414 Cardiopulmonary Status faced higher risks of mobility deterioration (25% vs. 9%; adjusted *p*‐value = 0.011). These findings emphasise the importance of cardiopulmonary assessments and effective transfer performance in improving functional mobility outcomes, with 94.9% achieving mobility improvements versus 5.1% with poor performance (adjusted *p*‐value = 0.011).

Check the Supporting Information 2 to see how to interpret the decision trees step by step.

## 6 Discussion

Trauma is one of the most neglected health crises of our time, making its prevention a priority. The World Health Organization underscores that preventing traumatic incidents from occurring remains the most effective strategy to reduce their frequency and mitigate their devastating consequences, even as timely treatment and appropriate management are essential for saving lives and improving patient recovery (Stewart et al. 2019; Sirajudeen et al. 2022; World Health Organization 2024). Trauma is a global issue but affects populations unevenly, both across and within countries, with vulnerable groups disproportionately impacted due to social determinants of health, such as the conditions in which people are born, grow, work, live and age (Stewart et al. 2019; Sirajudeen et al. 2022; World Health Organization 2024).

This study offers valuable insights into the demographic and clinical characteristics of trauma patients, further emphasising the importance of prevention and targeted interventions. A predominance of males in trauma cases reflects established evidence that men are more frequently involved in high‐risk activities. Motorcycle accidents, comprising 41.79% of all cases, emerged as a major cause of trauma, highlighting the critical need for prevention strategies. This is particularly relevant in rural areas of developing countries, where motorcycles are often the primary mode of transportation despite limited protective infrastructure and unsafe driving practices (Stewart et al. 2019; Sirajudeen et al. 2022; World Health Organization 2024; Santos et al. 2024).

The analysis of trauma severity scores and hospital resource utilisation reveals the significant burden of trauma on healthcare systems. The mean ISS of 35.06 indicates a severely injured population. Prolonged hospitalisations, including an average of 15.51 days in the ICU and 17.95 days in the ward, underscore the resource‐intensive nature of trauma care. Mortality and clinical outcomes were strongly associated with trauma scores: Patients who died had significantly higher ISS scores and TRISS averages compared to survivors. This demonstrates the predictive value of trauma scores for mortality and adverse outcomes, reinforcing the need for focused management strategies (Rapsang and Shyam 2015). Additionally, complications such as rhabdomyolysis, acute infections and fever were linked to poorer outcomes (Jakobsen et al. 2021; Abe et al. 2020; Pereira et al. 2023), emphasising the complexity of trauma care and the need for further research to refine trauma care strategies (Jakobsen et al. 2021; Abe et al. 2020; Pereira et al. 2023).

Clinical outcomes indicate that 69.15% of patients had the potential for full mobility recovery, 18.91% experienced no recovery and 11.94% achieved partial recovery. Patients with blunt trauma, who accounted for 88.05% of cases, had favourable recovery rates (84.08%), suggesting opportunities to enhance rehabilitation‐focused care pathways. In contrast, penetrating trauma patients experienced longer recovery periods and higher rates of permanent sequelae, consistent with findings in other studies (Nasirian et al. 2020). This underscores the importance of tailored rehabilitation and long‐term care planning.

Notably, the mortality rate observed in this study (6.97%) is lower than the typical 15%–32% reported in level 1 trauma centres (Candefjord et al. 2022), suggesting that targeted trauma care interventions were effective. Nursing strategies addressing mobility impairments played a key role in reducing mortality risk. Early and consistent mobilisation, including interventions such as range‐of‐motion exercises, transfer training and ambulation assistance, proved crucial in improving functional outcomes, shortening hospital stays and preventing complications like joint contractures (de Lima et al. 2020; Schafthuizen et al. 2024). These findings emphasise the value of proactive nursing interventions in improving patient outcomes.

This study also highlights the critical role of nursing outcomes (NOCs) in guiding care plans and decision‐making. Frequently identified outcomes included mobility, transfer, self‐care, nutrition and hygiene, reflecting progress in functional recovery. Walking was identified as a protective factor against mortality, while effective mobility and self‐care interventions reduced the risk of clinical deterioration and death. These outcomes, classified under the NOC Functional Health domain, emphasise the broader impact of nursing care on patient well‐being (Moorhead et al. 2018; de Lima et al. 2020; Schafthuizen et al. 2024). Additionally, early passive mobility exercises demonstrated significant benefits for critically ill and unstable patients. Key strategies included individualised programmes focusing on muscle strength, transfer training and pain management during exercises, alongside collaboration with rehabilitation teams and the use of assistive devices. These interventions not only enhanced recovery but also improved patient comfort and adherence to mobility‐related protocols (Moorhead et al. 2018; de Lima et al. 2020; Schafthuizen et al. 2024).

Furthermore, the integration of decision trees (DTs) in trauma care emerged as a promising approach to supporting clinical decision‐making. DTs transform complex patient data into interpretable pathways, assisting in risk stratification, prognosis estimation and intervention prioritisation. However, their reliability and validity depend on institutional protocols, patient populations and resource availability (Delebarre et al. 2022; Vera‐Salmerón et al. 2022). Ethical considerations, such as maintaining professional autonomy and safeguarding patient safety, are essential to ensure that nurses critically evaluate AI recommendations to ensure these tools enhance, rather than override, their clinical expertise (Delebarre et al. 2022; Vera‐Salmerón et al. 2022; Mohanasundari et al. 2023). DTs can also reduce the cognitive load on nurses, facilitating faster and more accurate decisions in high‐pressure environments (Delebarre et al. 2022; Vera‐Salmerón et al. 2022; Mohanasundari et al. 2023).

Moreover, decision trees, constructed using the CHAID algorithm, provided interpretable pathways that highlighted critical clinical indicators for risk assessment. While not designed for real‐time decision support, these models offered evidence‐based insights to prioritise nursing interventions. For example, the decision tree for mortality achieved a risk estimate of 10.9% with an accuracy of 89.1%, while the mobility decision tree achieved a risk estimate of 8.5% with an accuracy of 93.0%. These results demonstrate the robustness of the models (Refaeilzadeh et al. 2009; Jung and Hu 2015). The exclusion of NOCs with 0% or 100% prevalence ensured the inclusion of only relevant variables, enhancing the predictive accuracy of the decision trees.

To further ensure the reliability and clinical applicability of decision trees, complementary statistical approaches were used. Regression analysis validated the relationship between NOCs and patient outcomes, providing a statistical model for binary classification and confirming that improved walking and mobility reduce mortality risks. Additionally, K‐fold cross‐validation was employed to evaluate the models, ensuring stable performance and reducing overfitting. Key metrics used to assess model performance include risk estimates, which gauge the model's ability to generalise to unseen data, and accuracy, which measures the proportion of correct predictions. While accuracy offers a high‐level snapshot of the model's effectiveness, risk estimates provide deeper insights into its stability and performance across various scenarios (Refaeilzadeh et al. 2009; Jung and Hu 2015). This rigorous validation process enhances confidence in the clinical utility of the decision trees (Refaeilzadeh et al. 2009; Jung and Hu 2015). However, the lack of directly comparable nursing studies underscores the need for future research to establish benchmarks for these findings.

Finally, this study lays the groundwork for integrating AI tools like decision trees into clinical workflows. By enabling nurses to deliver targeted interventions without directly interpreting tree outputs, these tools align with the broader goal of improving patient outcomes while maintaining a human‐centred approach to nursing practice. Future research should focus on validating these tools across diverse environments and building larger, multi‐site databases to enhance their generalisability (Delebarre et al. 2022; Vera‐Salmerón et al. 2022; Mohanasundari et al. 2023).

## 7 Limitations

While the methodology provides a robust framework for adaptation in other environments, this study is limited by its focus on a local setting, which may affect the generalisability of the decision tree model across diverse clinical contexts, given variations in trauma care practices, patient demographics and healthcare infrastructure. Additionally, it should be noted that there was a limitation in the tests due to the differences in the number of participants in the categories of variables ‘death’ and ‘worsened mobility outcome’.

## 8 Future Research

Future research should explore the applicability, generalisability and validation of the model in different clinical contexts by expanding the sample size and patient characteristics and including data from multiple institutions and countries, thereby supporting evidence‐based improvements in trauma care. Additionally, future research is essential to better understand the positive and negative impacts of augmenting the nursing decision‐making process with AI tools, such as decision trees. This approach also highlights the need for updates in nursing education to incorporate AI literacy.

### 8.1 Conclusion

Decision tree‐based AI tools utilising standardised nursing terminologies can enhance nurses' clinical decision‐making regarding mobility and other effective nursing interventions. Through this novel application of decision trees within this context, of which no prior studies have explored, the transformative potential of decision trees further highlights the crucial role of early mobilisation, individualised rehabilitation strategies and assistive devices in the recovery process of critically ill trauma patients. Additionally, while our findings illuminate the great potentiality of AI, addressing ethical challenges is essential to ensure it complements rather than replaces clinical expertise.

## Author Contributions

R.C.F., K.D.‐L., S.M. and E.C.M.D. substantially contributed to conception and design, or acquisition of data, or analysis and interpretation of data. R.C.F., K.D.‐L., S.M., A.K., B.V.Z., L.A.C.C., M.T.S.L.B., and E.C.M.D. involved in drafting the manuscript or revising it critically for important intellectual content. All authors agreed to be accountable for all aspects of the work in ensuring that questions related to the accuracy or integrity of any part of the work are appropriately investigated and resolved and also gave the final approval of the version to be published. Each author should have participated sufficiently in the work to take public responsibility for appropriate portions of the content.

## Disclosure

This study received financial support from the Brazilian Coordination for the Improvement of Higher Education Personnel (CAPES). The authors declare that they have no conflicts of interest related to this research.

## Ethics Statement

This study was approved by the Research Ethics Committee of the State University of Campinas‐Brazil in 2019, under opinion number 3.587.784/2019, and registered with the Certificate of Presentation for Ethical Review (CAAE) number 13529619.3.0000.5404.

## Consent

Informed consent was obtained from all participants involved in the study.

## Conflicts of Interest

The authors declare no conflicts of interest.

## Supporting information


Supporting Information 1.

Supporting Information 2.

Supporting Information 3.

## Data Availability

The data supporting the findings of this study are available upon reasonable request from the corresponding author, Raisa Camilo Ferreira.
