# Supplementary material for: Decision Trees for Managing Impaired Physical Mobility in Multiple Trauma Patients
Source: J Adv Nurs. 2025 May 7;82(2):1359–70. doi: 10.1111/jan.17010 (PMC12810594; doi:10.1111/jan.17010)
Supplement: Supplementary file 2 — Supporting Information 2. [file JAN-82-1359-s001.docx]

**Supplementary file - How to interpret the Decision Trees figures**

**Decision tree for multiple trauma patients with a nursing diagnosis Impaired Physical Mobility (00085), in relation to the death outcome.**

**The structure:**

- **Root Node (Node 0)**: This is where the decision tree starts. It represents the initial condition where all patients are considered together.
- **Branches and Nodes**: Each branch splits the patients into different groups based on specific criteria (e.g., NOC). Each node shows the results for a subgroup of patients.

**Interpretation:**

**Node 0**: This node shows the overall likelihood of death among the patients.

- **Green (Yes)**: 7% of patients died (14 out of 201).
- **Blue (No)**: 93% of patients survived (187 out of 201).

**First Split**

The first decision point is whether the patient has the (NOC 0414) Cardiopulmonary Status (adjusted p-value = 0.004).

- **If Yes (Node 1)**:
- 20.8% died (5 out of 24).
- 79.2% survived (19 out of 24).
- **If No (Node 2)**:
- 5.1% died (9 out of 177).
- 94.9% survived (168 out of 177).

**Second Split**

For patients with a "No" the (NOC 0414) Cardiopulmonary Status (Node 2), the next factor is the (NOC 0210) Transfer performance (Node 2) (adjusted p-value = 0.033).

- **If No (Node 3):**
- 10% died (6 out of 60).
- 90% survived (54 out of 60).
- **If Yes (Node 4):**
- 2.6% died (3 out of 117).
- 97.4% survived (114 out of 117).

**Summarization of the results:**

- **(NOC 0414) Cardiopulmonary Status**: Patients with this NOC have a higher risk of death (20.8%) compared to those without (5.1%).
- **(NOC 0210) Transfer performance**: Among patients without the (NOC 0414) Cardiopulmonary Status, poor scores related to NOC indicators (NOC 0210) Transfer Performance further increases the risk of death (10% vs. 2.6%).

**Decision tree for multiple traumas patients with a nursing diagnosis Impaired Physical Mobility (00085), in relation to the worsened mobility outcome.**

**The structure:**

- **Root Node (Node 0)**: This is where the decision tree starts. It represents the initial condition where all patients are considered together.
- **Branches and Nodes**: Each branch splits the patients into different groups based on specific criteria (e.g., NOC). Each node shows the results for a subgroup of patients.

**Interpretation:**

**Node 0**: This node shows the overall likelihood of worsened mobility outcome.

- **Green (Worsened):** 10.9% of patients' conditions worsened (22 out of 201).
- **Blue (Others):** 89.1% of patients' conditions did not worsen (179 out of 201).

**First Split**

The first decision point is whether the patient has the (NOC 0414) Cardiopulmonary Status (adjusted p-value = 0.019).

- **If Yes (Node 1)**:
- 25% of patients' conditions worsened (6 out of 24).
- 75% of patients' conditions did not worsen (18 out of 24).
- **If No (Node 2)**:
- 9% of patients' conditions worsened (16 out of 177).
- 91% of patients' conditions did not worsen (161 out of 177).

**Second Split**

For patients with a "No" (NOC 0414) Cardiopulmonary Status (Node 2), the next factor is their (NOC 0210) Transfer performance (adjusted p-value = 0.011).

- **If No (Node 3):**
- 16.7% of patients' conditions worsened (10 out of 60).
- 83.3% of patients' conditions did not worsen (50 out of 60).
- **If Yes (Node 4):**
- 5.1% of patients' conditions worsened (6 out of 117).
- 94.9% of patients' conditions did not worsen (111 out of 117).

**Summarization of the results:**

- **(NOC 0414) Cardiopulmonary Status**: Patients with this NOC have a higher risk of mobility status worsening (25%) compared to those without (9%).
- **(NOC 0210) Transfer performance**: Among patients without the (NOC 0414) Cardiopulmonary Status, poor scores related to the NOC indicator (NOC 0210) Transfer Performance further increases the risk of the mobility worsening (16.7% vs. 5.1%)

**1. Describe the Overall Purpose**

Explain that this decision tree helps to understand the factors that affect whether a patient's condition worsens or remains stable.

**2. Explain the Structure**

- **Root Node (Node 0)**: Represents the initial condition where all patients are considered together.
- The first decision point is whether the patient has a specific cardiopulmonary status.
  - **If Yes (Node 1)**:
  - **If No (Node 2)**:

**Images**
