# Supplementary material for: Decision Trees for Managing Impaired Physical Mobility in Multiple Trauma Patients
Source: J Adv Nurs. 2025 May 7;82(2):1359–70. doi: 10.1111/jan.17010 (PMC12810594; doi:10.1111/jan.17010)
Supplement: Supplementary file 3 — Supporting Information 3. [file JAN-82-1359-s002.pdf]

OPINION EMBODIED BY THE RESEARCH ETHICS COMMITTEE(REC) OF BRAZIL

RESEARCH PROJECT DATA

Research Title: CROSS MAPPING, CLINICAL VALIDATION AND COSTS SURVEY OF THE NURSING INTERVENTIONS AND OUTCOMES FOR NURSING DIAGNOSIS “00085 IMPAIRED PHYSICAL MOBILITY” IN MULTIPLE TRAUMAS VICTIMS

Researcher: Raisa Camilo Ferreira

Thematic Area:

Version: 3

CAAE: 13529619.3.0000.5404

Institution Proponent: Hospital of Clinics from the UNICAMP- State University of Campinas

Main Sponsor: CAPES 001

OPINION DATA

Opinion Number: 3.587.784

Final Considerations at the discretion of the REC:

- The research participant must receive a copy of the Free and Informed Consent Form, in full, as signed (when applicable).

- The research participant has the freedom to refuse to participate or withdraw their consent at any stage of the research, without any penalty and without prejudice to your care (if applicable).

- The researcher must carry out the research as described in the approved protocol. If the researcher considers discontinuing the study, this must be justified and only carried out after analyzing the reasons for discontinuation by the approved REC. The researcher must await the REC’s opinion regarding discontinuation, except when unforeseen risk or harm to the participant is perceived or when verifying the superiority of a diagnostic or therapeutic strategy offered to one of the research groups, that is, only, if necessary, act immediately with the intention of protecting participants.

- The REC must be informed of all adverse effects or relevant facts that alter the normal course of the study. It is the researcher's role to ensure appropriate immediate measures in the event of a serious adverse event (even if it occurred in another center) and send notification to the REC and the National Health Surveillance Agency – ANVISA – together with their position.

- Any modifications or changes to the protocol must be presented to the REC in a clear and succinct manner, identifying the part of the protocol to be modified and its justifications and awaiting approval from the REC to continue the research.

- Partial and final reports must be presented to the REC, initially six months after the date of this approval opinion and until the end of the study.

-We remind you that according to Resolution 466/2012, item XI.2 letter e, “it is up to the researcher to present the requested data to the REC at any time”.

-The researcher must keep the research data in a physical or digital file under his or her custody. It is your responsibility for a period of 5 years after the end of the research.

This opinion was prepared based on the documents listed below:

Document Type	File	Post	Author	Situation
Basic information from the project	PB_BASIC_INFORMATION_DO_PROJETO_1352042.pdf	09/16/2019 01:14:31		Accepted
Others	TCLE_15_9ok.docx	09/16/2019 01:13:37	Ráisa Camilo Ferreira	Accepted
TCLE / Terms of Assent / Justification of Absence	TCLE_15_09_patients.docx	09/16/2019 01:11:51	Ráisa Camilo Ferreira	Accepted
Others	Letter_Response_15_9.docx	09/16/2019 01:11:39	Ráisa Camilo Ferreira	Accepted
Detailed project / Brochure Investigator	thesis_15_9.docx	09/16/2019 01:11:02	Ráisa Camilo Ferreira	Accepted
Title Page	sheet_de_rosto_cep.pdf	05/10/2019 20:30:22	Ráisa Camilo Ferreira	Accepted
Others	AttetoMatricula.pdf	05/10/2019 20:28:42	Ráisa Camilo Ferreira	Accepted

Status of the Opinion:

Approved

Requires CONEP Appraisal:

No

CAMPINAS, September 20, 2019

Signed by:

Renata Maria dos Santos Celeghini (Coordinator)
